# Medical College of Atlanta

**Published:** 1887-10

**Authors:** 


### THE MEDICAL COLLEGE OF ATLANTA.

This institution, whose annual course of lectures begins on the 5th instant, has filled the chair of obstetrics, vacated by the death of Professor Taliaferro, by the election of Dr. Virgil O. Hardon, of this city.

The college is to be congratulated on their fortunate selection. Dr. Hardon has not been long a citizen of Atlanta, but long enough to impress most favorably the profession and the public. His thorough training, his ripe scholarship, his gentlemanly bearing and high sense of professional honor give assurance that the distinction has been worthily conferred.

### ITEMS.

JNO. P. HAYNES, a Galveston, Texas, negro, has been appointed Demonstrator of Anatomy in Dartmouth Medical College.

ATLANTA MEDICAL COLLEGE.—The introductory address will be delivered October 5th, at the College, by Prof. Virgil O. Hardon.

IN long administration of bromides, as in epilepsy, no more of the remedy can be utilized by the system in combating the disease than that which will cause anæsthesia of the fauces.—*Ex.*

MEDICO-CHIRURGICAL COLLEGE, PHILADELPHIA, PA.—The introductory address will be delivered October 3rd, at the College, by Prof. W. F. Waugh.

THE last students of the St. Petersburg Medical School for Women has recently finished their last examinations. Thus this extremely useful institution has been successfully strangled by the reactionary clique which now governs the fates of the unhappy Russian people.—*Ex.*

PROPHYLAXIS OF CROUP.—Dr. Dumas believes that iodine, given internally, in quantities not exceeding eight drops *daily*, in orange-flower water, sweetened with syrup, with a little iodide of potassium, prevents attacks of croup.—*Medical Register.*

PLYMOUTH, PENNSYLVANIA, is threatened with a repetition of the dreadful epidemic which devastated it in 1885. It is stated by physicians that the whole community is threatened with an epidemic of typhoid fever from the careless disposal of household wastes. There are thirty cases of fever there now.—*Ex.*

A CENTENARIAN DYING IN HARNESS.—Dr. S. P. Neklevitch has just died at Lozki, in the Novogrudsky District, Russia, in his 109th year. His death occurred quite suddenly, as, about a quarter of an hour earlier, he wrote a prescription for one of his patients.—*Ex.*

AT a recent meeting of the American Gynæcological Society in New York city, Dr. Robert Battey, of Rome, Ga., was elected to the office of President of the Society. This Society is one of the most exclusive of the medical organizations of this country, and an election to its presidency is one of the highest honors to which medical men of America are eligible, yet we cannot help feeling that the Society is honored by its newly-elected President quite as much as he is honored by his high position.

A CORRESPONDENT contributes a note from his practice which tends to show that grief may be very poignant, and yet not obliterate the sense of the beautiful. In the case of a patient suffering from meningitis, the head was ordered shaved. In a few days the man died, and his brother, on reporting the demise to the doctor, said: "It may be all right, doctor, to shave a man's head for that disease, but poor George makes a h— of a looking corpse."—*Medical Age.*

IN one of the hospitals during the war there was a young soldier who happened to be good-looking and near the door. The majority of amateur nurses that came into the room wanted to do something for him. A young lady came into the room one day and said, "Can I not do something for you?" "Perhaps," he replied. "Can't I wash your face?" "Yes, if it will give you any pleasure, but you are the thirty-seventh that has done it to-day."—*Ex.*

CARBOLIC ACID IN OTITIS.—Dr. Hartamann during the last year has treated moderate otitis with instillations of several drops of a solution (1 in 10) of carbolized glycerine. The results were excellent. Pain instantly disappeared and the progress of the affection was checked. In cases where effusion existed, the relief obtained was equally great. M. Rohrer, who confirms M. Hartamann's statements, recommends a solution of 20 per cent.—*British Medical Journal.*

A MYOPIC HORSE.—One of the attractions, and an observed of all observers, of the Clinton market place in Boston not long since, as we learn from a daily paper, was a horse wearing spectacles. The animal was very near-sighted, and an oculist took the necessary measurements and had a pair of concave glasses made for it. Unfortunately the report does not state just how the animal was examined, whether he can count fingers at ten feet, or what Jaeger test type he can now read.—*Jour. Amer. Med.*

THE CURABILITY OF CIRRHOSIS OF THE LIVER.—At a recent meeting of the French Academy of Medicine, M. Lancereaux read some observations which prove the curability of this disease by a milk diet and the administration of iodide of potassium. The best results are obtained in the atrophic variety. Hypertrophic cirrhosis is more difficult to cure. When icterus exists, and especially when it is very marked, death is almost certain. The duration of the treatment varies with the gravity and the duration of the disease. Generally by the end of fifteen days, a decided amelioration takes place. The treatment should be continued for a long time, at least a year.—*Journal de Médecine.*

DOUBLE URETHRA IN THE MALE.—At a recent meeting of the "Verein der Aerzte in Steiermark" (Society of Physicians of Styria), Prof. Lipp brought forward a man in whom a double urethra was to be seen. The patient, æt. 25, had been admitted into the hospital owing to acute gonorrhœa, and on examination a second opening, which was surrounded by a limbus, and through which one could introduce the sound into a canal of the size of 17 c. m., being coated with mucous membrane (basement epithelium), was found to be present over the opening of the urethra near the corona glandis. The canal went as far as the posterior part of the symphysis; the process was in both ureters a purulent one.—*Medical Press and Circular.*

A POST-MORTEM IN THE OLDEN TIME.—Describing the sufferings and last illness of that famous Caroline divine, Dr. Hammond, his biographer mentions that, in addition to several attacks of suppression of urine, he also suffered from repeated and protracted attacks of epistaxis, the last of which brought matters to a crisis with him ; he says that "the dead body being opened, on the 25th of April, 1660, the principal and vital parts appeared sound; only the right kidney, or rather its remainder, which exceeded not the bigness of an egg, was hard and knotty, and in its cavity, besides several little ones, a large stone of the figure of an almond, though much bigger, whose lesser end was fallen into the ureter, and as a stopper closed it up; so that it is probable that the kidney has been for divers years in a manner useless. The other kidney was swollen beyond the natural proportion, otherwise not much decayed; but within the ureter four fingers breadth, a round white stone was lodged, which was so fastened in the part that the physician with his probe could not stir it, and was fain at last to cut it out, and so exactly it stopped the passage that upon dissection, the water before enclosed gushed forth in great abundance, from whence it appeared perfectly impossible for art to have ennobled itself in the preservation of this great person." Whether nephrectomy, or that lesser procedure, nephro-lithotomy, would have been of any avail at an earlier period of his illness is a question we must leave to the advocates of those measures, but they probably would not, and it is surprising that our patient lived with these complications to the age of 55.—*Medical Press and Circular.*

The following resumé of a paper read before the Section of PHYSIOLOGY, NINTH INTERNATIONAL MEDICAL CONGRESS, Washington, D. C., is taken from the Philadelphia *Medical Register*, of September 9th, 1887:

A NEW THEORY OF LIFE FORCE.—Origin of Nerve-Force from the Electro-magnetic Condition of the Oxygen of the Atmospheric Air, through a Function of the Red-Blood Corpuscles not hitherto so designated. By Wm. Abram Love, M. D., of Atlanta.

Dr. Love laid down as the basis of his paper, the fact first given us by Michael Faraday, that the magnetic tension of oxygen varied from 17.5 to as low as 3.4. He then spoke of the development of ozone by the passage of a current of electricity through a volume of oxygen, and gave a tabulated statement of the relative tensions of atmospheric air and of oxygen under different atmospheric pressures, showing the increase or decrease of the tension, in the ratio of the increase or decrease of the pressure, and proving that the magnetic tension of one atmosphere of pure oxygen was equal to atmospheric air under a pressure of five atmospheres, or sixty pounds to the square inch. He then passed to the consideration of the function of the red-blood corpuscles, as bearers of oxygen from the atmospheric air through the lungs to the tissues at the end of the peripheral systemic circulation.

Dr. Love holds the position that oxygen, among the gases, is, to electricity or magnetism, what iron is among the metals. He holds that the red-blood corpuscles in becoming charged with oxygen in the process of respiration, become charged at the same time with electricity or magnetism, and that in the systemic circulation this electric or magnetic force is discharged by the red-blood corpuscles, transferred to the end organs or terminals of the nerves, and is carried thence to the nerve-centers and deposited in them as the store-houses of the nervous system to be modified or transferred into nerve-force for future use in carrying on vital action. He then adduces evidence, in established facts and the results of experiments, to prove that the effect of the inhalation of oxygen or compressed air produces severe and dangerous symptoms, affecting the nervous system. He then gives the results of the inhalation of condensed oxygen by animals, showing that it produces convulsions resembling those arising from tetanus, or in epilepsy, and strongly resembling the toxic action of strychnia or picric acid. He brought in evidence the fatal results to the workmen who worked under the pressure of two or three atmospheres in the caissons constructed to be worked under pneumatic pressure, in digging out the foundations and building the piers for the bridge across the Mississippi at St. Louis, where the men died rapidly with symptoms resembling in some respects cerebro-spinal meningitis. He concludes that if the surcharge from increased magnetic tension, by reason of compressed atmospheric air or oxygen breathed, produces such powerful effects, stimulating the nervous system into dangerous and fatal conditions, giving such an excess of action pathologically as to produce death, we must necessarily arrive at the conclusion that at a *normal tension* the same character of changes must take place, giving *physiological nervous functional action.* He then applies these facts to the establishment of the principle for which he contends:

1. That oxygen, like iron, is capable of holding within itself a magnetic force, varying in intensity, from the highest charge found in compressed ozone (dangerous from its excess of tension) to the low tension in which it will combine in equal equivalents with carbon—$C_2$ $O_2$, olefiant gas—carbonic oxide—a low form of gas that fixes itself in the corpuscle to the destruction of its vital function.

2. That the red-blood corpuscles, in becoming arterialized, become at the same time charged to a degree commensurate with the tension of the respired air, or to saturation.

3. That this charge is carried to the peripheral systemic circulation, where, on the carbonization of the blood, it is discharged into the end organs of the nerves, supplying the surrounding tissues, and deposited in, the nerve-centers, the store-houses of nerve-force for future use, or, so much as may not be applied directly to the tissues, as in the muscular tissue.

Dr. Love does not believe in the teachings that nerve-centers have the power to generate or *originate* nerve-force. He alludes in the course of his paper to the effects of rarified air and the reduction of tension and vital force, to the symptoms of *mal des Montagnes* and *balloon sickness*; the neurasthenic and anæmic condition of patients induced by respiring the rarified air of the great Mexican plateau, and takes the position that a reduction of pressure and a reduction of tension in the air respired *is equivalent to a reduction of the red-blood corpuscles in the blood.*

In the course of his paper, Dr. Love alludes to the question of *malaria* and the effects of marsh drainage in mid-summer.

He contends that it results in the devitalization of the air by diverting the oxygen and its tension to the work of carrying on the process of decomposition and combustion, which is done at the expense of the oxygen and tension force of the surrounding atmosphere, and that here we have a vitiated atmosphere, not by reason of what has been added to it, but by reason of what has been taken from it. This atmospheric condition deprives the blood corpuscles of the means with which to carry on their functional action; the blood is diminished in its vital force; the organs fail in their functions; the secretions become vitiated; the nerve-force becomes depressed by reason of want of supply in the weakened condition of all the forces and functions; the diurnal changes assume a prominence, and some form of paroxysmal disease, with its well-known periodicity, presents itself.

Dr. Love contends that the reasons for advised delays, in swamp or marsh drainage, to the approach of winter, are, or should be, that their combustion would be less rapid; the oxygen tension would be increased by reason of the development of ozone; hence the vital force of the atmosphere would not be reduced to a degree calculated, by the division and the diversion, to endanger health and life.

Dr. Love, in conclusion, stated that in presenting this paper he had done so *in a search after truth.* He asked for the ideas therein presented, a fair, faithful, unbiased and unprejudiced investigation, expressing the hope that those who found them would frankly show the seeming errors and kindly point the better way.

IN a timely and thoughtful paper on "Municipal Government," to be published in the October number of *Scribner's Magazine*, Mr. Gamaliel Bradford states, with novelty and force, reasons for his conclusion that the true remedy for the evils now existing in city government will be found in concentration of responsibility.

THE DANGERS OF FOOT-BALL.—From a profusely-illustrated article in the October *Century*, by Alexander Johnston, we quote the following: "The game is as safe as any out-door game can well be, provided it is played with the careful preparation and training which are the rule in the larger colleges. It is a dangerous and unfit game when men undertake to play it without such preparation and training. In the season of last year two fatal accidents were reported; both occurred in colleges which were attempting to play the game as it is played by the leading teams, without any of the preparation which they find an essential. The writer, who has been in the habit of attending the regular games of the college with which he is connected, has felt under obligations to be equally consistent in attending the daily practice games of the men in order to watch the preliminary training; and he must confess to a great respect for the good sense and the good management of the under-graduates who have the matter in charge. The 'University team' is selected provisionally; it is pitted daily against a second, or 'scrub,' team of somewhat larger numbers; both teams are kept under careful training and supervision; the playing is made short and as gentle as possible at first, until the men begin to become 'hard;' the playing is then gradually lengthened and made more severe, as the men become able to endure it, and, by the time the season comes to its last game, the players are able to endure with impunity treatment which would be dangerous to men who are 'soft,' or out of condition. After the first few weeks are over, and serious playing has begun, men who have not yet played are not encouraged, or, in extreme cases, even allowed, to play on the 'scrub' team; the managers think it inadvisable to run any risk. The players are not only brought to a point of physical condition, which makes it a pleasure to watch them; they are taught how to fall, when a fall is inevitable, in such a way as to retain control of the ball without hazarding a broken bone or dislocation. When the closing games come on, the player can take what seems to the spectator a frightful fall, not only without a bruise, but so skillfully that it is regularly necessary for his opponent to 'hold him down' lest he rebound and take to his heels again. The preliminary practice games can hardly be more severe elsewhere than at Princeton; and yet the writer has never seen a serious accident occur there. An accident may occur, of course, and will give no warning of its coming, but its coming has been put as far as possible out of the range of probability. But if men in other colleges wished to play foot-ball, as should be the case, they must not ignore the systematic course of preparation, take the final playing of a well-trained team as a model, and attempt to imitate it. It is from such folly that the recurring accidents in foot-ball come. With good physical condition in the players, the requisite training and suitable grounds, the game is not only one of the best of out-door sports, but one of the safest."

OURSELVES AS OTHERS SEE US.—The New York *Commercial Advertiser*" lately published the following article: "The International Medical Congress, now in session at Washington, is composed of unusually learned and able and intelligent men. They have dived deeply into the hidden nature of things; they have studied hard; they have investigated closely substances that are too infinitesimal to be apprehended by the unassisted senses; they are fired by an insatiable ambition to know more and more. Why is it, then, that they seem to know so little? They write whole libraries on otology and gynæcology and laryngology and bacteriology, and various other ologies, which the average educated man can neither define nor spell, and can hardly manage to pronounce; they have more drugs and surgical instruments, and healing appliances of all sorts, than there are hairs on a normal head; and still they cannot, except in rare instances, keep a man in good health, even if the patient does exactly as he is told to do. We do not require the impossible. We must all die, and no one will blame the doctors for failing to preserve life indefinitely or for being unable to avert fatal consequences in the case of a severe accident or disease. The question is not so much 'Why don't we get well?' as 'Why can't we keep well?' People are seldom downright ill, but they are perpetually ailing. Why cannot the doctors stop this? It is fair to assume that they cannot, because they certainly do not. The preservation of health is largely a matter of personal habits. It depends greatly on our way of eating, drinking, and taking care of the body generally. But there is no subject on which the doctors differ more hopelessly than on this. Some recommend tea and coffee; others denounce them as poisons. Some permit tobacco and alcohol in moderation; others forbid them entirely. Some say that pork and ham, though hard to digest, are still useful foods; others declare that no pig meat or anything derived from the pig is fit to be eaten. Some advise fruit only before breakfast; others only after dinner. Some depreciate the vegetables, others extol them as a main reliance. Some counsel bathing in cold water; others in warm or tepid. Some say we must sleep with the windows open always; others limit this injunction to the summer season. It is scarcely an exaggeration to say that no two doctors will give you the same instruction as to the commonest physical habits of life. Now, messieurs the doctors, it is all very well for you to raise men from the dead, and to cure violent diseases, and to be letter-perfect on sarcoma of the larynx and the bacteriology of aural furuncles; but what we want to know is, What shall we eat? and what shall we drink? and wherewithal shall we be clothed? The Gentiles are seeking after all these things, and they propose to find them out, too. It surely ought to be possible for the doctors, at this late day, to tell definitely whether coffee is a food or a poison, and how a sleeping apartment ought to be ventilated. It will not do to fall back on the old excuse that what is one man's food is another man's poison. Constitutions differ, doubtless, but not to that extent. Any particular chemical substance—nicotine or alcohol, for example—has a certain fixed, unvarying effect on every normal mucous membrane, and that effect must be either beneficial or deleterious to the membrane and the system. Which is it, messieurs the doctors?"—*New York Medical Journal.*

ATROPINE IN HÆMOPTYSIS.—Dr. Haussman prescribes subcutaneous injections of atropine as a last resort in cases of serious hæmoptysis. He cites three cases in which this remedy produced excellent results. The first was that of a patient who had serious hæmoptysis twelve times in six days; three milligrammes of sulphate of atropine were injected; there was no recurrence of hæmoptysis. The same result was obtained in the second patient, in whose case the administration of turpentine preparations and injections of ergotin had produced no improvement. In the third case a patient sufferieng from repeated hæmoptysis was cured by two subcutaneous injections of three milligrammes of sulphate of atropine.—*British Medical Journal.*

NEW REMEDIES FOR THE RELIEF OF MORPHIA CRAVING.—Dr. Oscar Jennings (*The Lancet*, June 25, 1887) announces the discovery of certain drugs which, he says, will enable any morphia *habitué*, earnestly desirous of leaving off his intemperance, to carry the process of weaning to a successful issue. All morphia *habitués* have from time to time a maudlin, hysterical desire to give up the practice, but this vanishes at the first feeling of discomfort. For such cases there can be no suppression without restraint, and a result so obtained is very unsafe for the future. By the help of sparteine and nitro-glycerine, however, we are told that the patient can get relief from his craving until the system has accommodated itself to the absence of morphia. The mode of action of the drugs mentioned is as follows : Professor Ball and Dr. Jennings have shown that the heart in morphinomania is in a very depressed state, and that there is a general ischæmia of the circulation. Morphine relieves this, and with it the morbid craving. But sparteine and nitro-glycerine do the same, as shown by sphygmographic tracings. "The morphia craving," says Dr. Jennings, "is a complex suffering, at the same time cerebral and peripheral, each factor of which can be relieved by appropriate treatment. The organic distress may be soothed by sparteine, which recovers the heart in the same way as a hypodermic injection of morphia. The cerebral craving (or yearning), often the more painful of the two, corresponds to a want of habitual congestion. This can be arrested by nitro-glycerine, which gives rise to the same kind of sensation in the morphia *habitué* deprived of his stimulant as the hypodermic injection. It acts probably by inhibition *à distance* rather than by essential sedation of the nervous centres." There have been many remedies for the morphia habit, from cinchona bark and Scotch oats to cocaine. All have been, more or less, cardiac and brain stimulants, and it is not easy to understand why sparteine and glonoin should act with any special efficiency. Certain it is that there is no specific for the opium habit.—*N. Y. Medical Record, Aug. 27th, 1887.*

DETERMINATION OF THE PURITY OF IODOFORM.—The most contradictory statements are made in regard to this valuable medicine. In some hospitals its use has given the most brilliant results, while in others numerous accidents have occurred from it. The attention of the Congress of German physicians having been called to this subject, a practical method is proposed for the verification of the drug. It consists in agitating the iodoform with distilled water, filtering and treating with an alcoholic solution of nitrate of silver. If, at the end of twenty-four hours, it forms a precipitate, it is a sign that the specimen contains soluble foreign substances, for when it is pure, the only result of the mixture is a grayish-white tint. All specimens of iodoform which reduce nitrate of silver cause poisoning. If the iodoform is pure, but kept too long, it acquires, under the action of air and light, toxic properties.—*Journal de Médecine.*

DR. L. E. BORCHEIM, of this city, died by his own hand at the Kimball House, September 11, 1887. He was a physician of acknowledged ability, and had acquired a lucrative practice. For several months he had been in bad health, and it is said to have become addicted to the opium and morphine habits. Despondency over his physical condition was undoubtedly the cause of his rash act. He was unmarried and about thirty years of age. He graduated in New York, served as interne in Bellevue Hospital, and was for a time a surgeon in the United States army. He had practiced medicine in Atlanta for about five years.

A COURT PHYSICIAN BEHEADED.—The Ameer of Afghanistan suffered not long ago from a boil on his neck, and sought relief at the hands of his court physician. The latter ordered some sort of a salve to bring the boil to a head. Unfortunately the application caused the august patient considerable pain, and after a night of torture he sent for his physician and had him decapitated. Evidently the physician to the Ameer does not hold a sinecure.—*Med. Record, Sept. 3, 1887.*

THE OBSTETRICAL ADVANTAGES OF OTTER TAIL COUNTY, MINNESOTA.—Otter Tail, one of the frontier counties of Minnesota, bids fair to rival the Rotunda Hospital or the Allgemeine Krankenhaus, of Vienna, as a resort for aspirants for obstetrical advantages and greatness. Dr. T. G. Hutton, of Fergus Falls, writes us that there are now in that county living quadruplets one week old, living triplets eight months old, living twins born of a sixteen-year-old mother, and a child twelve months old whose mother is now only fourteen and one-half years old. It may be added, for the benefit of those who may hesitate between Vienna and Otter Tail county, that the latter place is just as accessible as Vienna, and the cost of living is less. The only reasonable objection to the frontier county is that the vernacular is English; nevertheless, it contains, we believe, a large number of Germans.—*Journ. Am. M. Assoc.*, Sept. 3, 1887.

DR. EUGENE FOSTER, a prominent physician of Augusta, was in the city during the past week.

DR. J. EMMETT BLACKSHEAR, of Macon, has moved to Savannah, and will make that city his future home.

DR. N. O. HARRIS has been appointed as Assistant to the Chair of Obstetrics and Gynæcology in the Atlanta Medical College.

DR. WILLIAM S. KENDRICK, of this city, a first-honor graduate of the Atlanta Medical College, of the class of 1874, has been appointed Proctor in that institution to fill the vacancy caused by the death of the late Dr. James A. Gray.

THE *Climatologist* is the name of a new journal to be started in October. It is to be a quarterly of forty-eight pages, and will be published at fifty cents a year. Dr. Geo. H. Rohe, of Baltimore, is to be the editor. From his interest in this subject and former experience in medical journalism, we shall expect a very superior journal.

QUITE an interesting episode occurred at the Congress during the distribution of the badges. One of the doctors, with his wife by his side, having received his badge, was placing it on his coat when his wife, struck with its beauty, asked if she couldn't have one also. The distributor wanted to know if she had a title which entitled her to one. She replied immediately that it was m-a-m-a. A broad grin went around the group of faces, and it is needless to say that the lady got the badge.—*Medical Review.*

A "HEADQUARTERS" will be fitted up in the main building at Piedmont Exposition by Messrs. Parke, Davis & Co., of Detroit, Mich., for the reception and convenience of physicians and druggists and their ladies. Make it your post-office and telegraphic address, and write your friends to meet you there.

THE yearly income of the *British Medical Journal* is said to be $165,000.

THREE internes of a Newark City hospital have been arrested for mistaking a comatose patient for a dead drunk when he was suffering from tumor of the brain, as demonstrated by the post-mortem examination. If all doctors that have made similar mistakes had been treated in a similar manner, the number of active practitioners would be slightly less than now.—*Ex.*

A CORRESPONDENT of the *Boston Medical Journal* relates a case in which a young man began and completed his medical education within nine months, obtaining a diploma from a celebrated medical school. We presume that many other physicians could relate similar stories. Such is one of the ways by which the profession of medicine is recruited with persons who will do it honor.—*Ex.*

CHOLERA IN NEW YORK.—Four deaths from cholera and twenty-three cases have been reported as occurring in New York during the past month. They were passengers of the steamship, Alesia, which sailed from Naples September 3, and arrived in New York Septembrr 23. The Alesia has been sent to quarantine, and all precautionary measures taken to prevent the spread of the disease.

CHOLERA IN INDIA.—Cholera is extensively prevalent in India this season. The death-rate from this cause in Calcutta during the winter was unusually high, and during May, June and July, Bombay, the North West Provinces and Oude have suffered severely. The most ordinary sanitary precautions, with regard to water supply especially, are systematically neglected, and there seems to be a want of an appreciation on the part of the government of the value of systematic inspection, and the enforcement of simple methods of securing drinking water from contamination by natives who do not hesitate to bathe and wash therein.—*British Medical Journal.*

COCAINE IN DISLOCATIONS.—So many papers and notes have been contributed on the almost endless uses of cocaine that one hesitates before bringing under the notice of his brethren any further application of the drug.

However, the other day I found it useful in a way that was new to me, and venture, even at the risk of treading ground already covered, to send you an account of the case.

A mason fell off a platform where he was at work and dislocated his elbow very badly, the skin being so thinned by stretching as almost to let the bones protrude. His pain was most intense, and he could not tolerate the least handling of the limb. Five minims of an 8 per cent. solution of cocaine, injected deeply near the head of the radius, produced complete local anæsthesia, and relaxed all muscular tension, so that the dislocation was readily reduced absolutely without pain.

I may add also that recently I was able to extract a fishing-hook from a little lad's finger, backwards, without his feeling it at all, after injecting a few drops of cocaine alongside the hook.—*Geo. C. Kingsbury, M. D., in British Medical Journal.*

LONDERER reports a case of prostatic hypertrophy, in which the crushing of a small stone was rendered impossible on account of the enlargement, and consequently the median section was made. In the course of the operation a portion of the prostate gland was accidentally removed, and from that time to the present, fifteen months after the operation, the patient has had none of the previous difficulty in passing urine. It is therefore suggested that removal of a portion of the prostate be resorted to as an operative procedure for prostatic hypertrophy with difficult passage of urine.—*Medical Review.*

TRANSMISSION OF TYPHOID FEVER BY AIR.—At a recent meeting of the Société Médicale des Hôpitaux, M. Féréol read an account by M. Devalz, of Eaux-Bonnes, of an epidemic of typhoid fever, in which the author discovered an instance of the transmission of the morbid germ by the air. A patient, presenting the first symptoms of typhoid fever, arrived at an hotel at Eaux-Bonnes. She recovered in four weeks, but the three daughters of the hotel-keeper were successively attacked with the malady. There was no other case of typhoid fever in the town, which is plentifully supplied with pure spring water. Bacteriological examination showed this water to be free from suspicious organisms. During the treatment of the first patient, no disinfecting measures had been taken; fæcal matters were emptied into the water-closets of the hotel; the door of these privies opened on to an ill ventilated passage, in which the daughters of the hotel-keeper slept. Their room, which contained only one door and window, both opening on to the passage, was at only a yard's distance from the closets.—*British Medical Journal.*

THE medical societies are of great value to those members who work and compare results; they are valuable to the practitioner who is too modest to speak and who has not learned to write. They are like the literary societies in college. They create community of knowledge and unity of purpose. They do more toward diffusing practical information than any school, however perfectly equipped for teaching, yet the outsiders proclaim against societies, and find, after awhile, that they are like poor Othello, whose occupation had gone forever.—*Progress*

HYDROPHOBIA.—The severe dog law in Bavaria has been the means of virtually stamping out hydrophobia in that country. During the last seven years there have been only three deaths from hydrophobia out of a population of 6,000,000. Every dog in the country is bound, upon pain of instant death, to bear upon his collar a metal tally, upon which is inscribed his number upon the register of his district. The color and shape of this tally, which is really the dog's passport, are changed every year, and the police are thus able to see at a glance if a dog is "in order." Once a month all dogs have to be examined by a veterinary surgeon, and if they are not in good health, they are detained in a dogs' hospital until they recover. If an animal changes hands, the transfer must be at once notified to the police, and any breach of the regulation, even a delay of a few days in the payment of the tax, is visited by a heavy fine.—*Medical Register.*

HOW TO MAKE A MAN!

| | | |
|---|---|---|
| ℞ | Ferri | ℥j ʒij |
| | Sodii | ℥ij ʒij |
| | Potassii | ℥ij |
| | Sulphuris | ℥ij ʒij |
| | Phosphor | ℥xxvi |
| | Fluorini | ℥iij ʒij |
| | Chlorini | ℥xxvj |
| | Nitrogeni | iv lbs |
| | Hydrogeni | xv lbs |
| | Calcii | iij lbs |
| | Carbonis | xlvij lbs |
| | Oxgeni, ad | 168 lbs |

Misce bene "secundum artem" et adde "Vitæ" q. s. ut fiat homo.—*Medical Review.*

SICK MAN—Am I to take all that medicine? Wife—Yes, all of it. Sick man—There's enough in that bottle to kill a mule. Wife—No, there isn't, John, or the doctor wouldn't have prescribed it.—*Puck.*

